# The role of neutrophil gelatinase-associated lipocalin and iron homeostasis in object recognition impairment in aged sepsis-survivor rats

**DOI:** 10.1038/s41598-021-03981-7

**Published:** 2022-01-07

**Authors:** Yoshikazu Nikaido, Yoko Midorikawa, Tomonori Furukawa, Shuji Shimoyama, Daiki Takekawa, Masato Kitayama, Shinya Ueno, Tetsuya Kushikata, Kazuyoshi Hirota

**Affiliations:** 1 Department of Frailty Research and Prevention, Hirosaki University Graduate School of Medicine, Hirosaki, 0368562 Japan; 2 Department of Anesthesiology, Hirosaki University Graduate School of Medicine, Hirosaki, 0368562 Japan; 3 Department of Neurophysiology, Hirosaki University Graduate School of Medicine, Hirosaki, 0368562 Japan

**Keywords:** Sepsis, Iron, Neurochemistry

## Abstract

Older adult patients with sepsis frequently experience cognitive impairment. The roles of brain neutrophil gelatinase-associated lipocalin (NGAL) and iron in older sepsis patients remain unknown. We investigated the effects of lipopolysaccharide-induced sepsis on novel object recognition test, NGAL levels, an inflammatory mediator tumor necrosis factor-α (TNFα) levels, and iron ion levels in the hippocampus and cortex of young and aged rats. The effect of an iron chelator deferoxamine pretreatment on aged sepsis rats was also examined. Young sepsis-survivor rats did not show impaired novel object recognition, TNFα responses, or a Fe^2+^/Fe^3+^ imbalance. They showed hippocampal and cortical NGAL level elevations. Aged sepsis-survivor rats displayed a decreased object discrimination index, elevation of NGAL levels and Fe^2+^/Fe^3+^ ratio, and no TNFα responses. Pretreatment with deferoxamine prevented the reduction in the object recognition of aged sepsis-survivor rats. The elevation in hippocampal and cortical NGAL levels caused by lipopolysaccharide was not influenced by deferoxamine pretreatment. The lipopolysaccharide-induced Fe^2+^/Fe^3+^ ratio elevation was blocked by deferoxamine pretreatment. In conclusion, our findings suggest that iron homeostasis in the cortex and hippocampus contributes to the maintenance of object recognition ability in older sepsis survivors.

## Introduction

Sepsis is a form of multiple organ failure that is associated with high mortality and caused by dysregulation of innate immune responses to infection^1^. Older adult patients with sepsis often experience cognitive dysfunction, including sepsis-associated encephalopathy (SAE)^2,3^. The pathogenesis of SAE involves various processes, such as neuroinflammation, oxidative stress, and disruption of the blood–brain barrier (BBB)^4^. Peripheral proinflammatory cytokines such as tumor necrosis factor α (TNFα) and interleukin-1β induce impairment of BBB integrity and deliver inflammatory signals to the central nervous system (CNS)^4^. Furthermore, sickness behavior and neuroinflammation induced by bacterial endotoxin lipopolysaccharide (LPS) are exacerbated with aging^5,6^. Despite progress in our understanding of the pathogenesis of SAE, we are still unable to prevent SAE. Thus, SAE remains a critical issue related to postoperative and intensive care unit care.

Acute-phase protein neutrophil gelatinase-associated lipocalin (NGAL) is an inflammatory mediator and its levels are clinically correlated with sepsis severity^7^. NGAL is also involved in iron delivery to and export from cells via a transferrin-independent mechanism^8^. Systemic LPS injection increases peripheral and brain NGAL concentrations^9,10^. Under pathological conditions, NGAL contributes to the dysregulation of iron metabolism, glial activation, and neuronal death^11–14^; however, some studies have reported that NGAL has neuroprotective functions^15–17^. Our recent microarray study found that the iron chelator deferoxamine (DFO) can attenuate NGAL mRNA expression upregulation induced by chronic diazepam treatment in the mouse hippocampus and cortex^18^. Serum iron and ferritin levels are positively correlated with sepsis severity and mortality^19^. Iron in the CNS plays essential roles in numerous biological processes such as mitochondrial respiration, protein synthesis, and cellular metabolisms; however, iron accumulation in the cortex and hippocampus is a risk factor for neurodegenerative diseases^20^. Ferric (Fe^3+^) and ferrous ions (Fe^2+^) are sources of reactive oxygen species and contribute to the aggregation of Amyloid-β, Tau, and α-synuclein^20^. These findings suggest that NGAL and iron metabolism might have therapeutic potential for cognitive dysfunction associated with neuroinflammation and neurodegeneration.

In this study, we investigated the effects of systemic LPS injections on brain NGAL levels, TNFα levels, iron ion levels, and object recognition in young and aged rats. Furthermore, we examined whether DFO treatment could ameliorate LPS-induced cognitive dysfunction in aged rats.

## Results

### LPS induced lethal sepsis and object recognition deficit in aged sepsis-survivor rats

**Figure 1:**
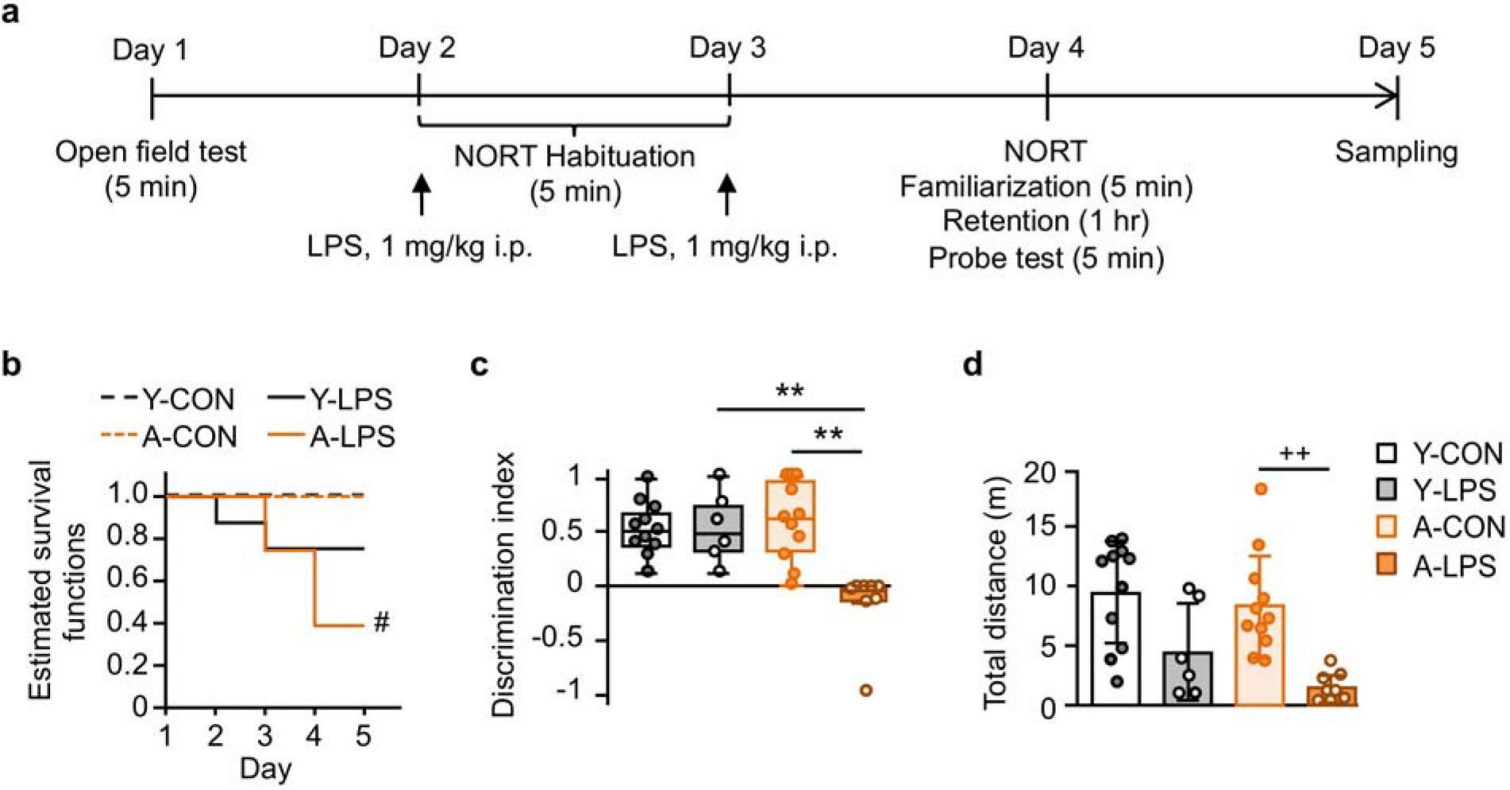
Dual lipopolysaccharide (LPS) challenge induced lethal sepsis and object discrimination impairment in aged rats. (**a**) Schematic representation of the experimental protocol. Young and aged rats were assigned to control or 1 mg/kg LPS intraperitoneal injection groups after being subjected to the open field test (Y-CON group, n = 11; A-CON group, n = 11; Y-LPS group, n = 8; A-LPS group, n = 20). Y-LPS and A-LPS group rats were administered LPS after each novel object recognition test (NORT) habituation session. On Day 4, survivor rats were subjected to the NORT. (**b**) The Kaplan–Meier survival curves showed that the survival rate of the A-LPS group was significantly lower than that of the A-CON group. (**c**,**d**) In the NORT, the discrimination index and locomotor activity of the A-LPS group were significantly reduced compared with those of the A-CON group. There were no significant differences in the survival rate and the NORT performance between Y-CON and Y-LPS groups. The behavioral data are presented as the median ± IQR or mean ± SD. Y-CON, n = 11; Y-LPS, n = 6; A-CON, n = 11; A-LPS, n = 8. Log-rank test with Bonferroni correction, ^#^P < 0.05, A-CON vs. A-LPS; permuted Brunner-Munzel test with Bonferroni correction, **P < 0.01, effect size p̂ ≥ 0.96; Welch’s ANOVA [F_3,14.1_ = 17.7, P < 0.001, ω^2^ = 0.73] and post-hoc Bonferroni corrected Welch’s *t*-test, ^++^P < 0.01, Hedge’s g = 2.08.

We first investigated the effects of LPS-induced sepsis on survival and object recognition in young (n = 19) and aged rats (n = 31; Fig. 1a). To confirm the effects of aging on the baseline anxiety state of the rats, we evaluated the anxiety-related behavior in the open field test (OFT) before LPS injections. There was no significant difference in anxiety-related behavior between groups [time spent in the center area, median (inter-quartile range, IQR): young, 2.9 (0.0–10) sec; aged, 2.4 (0.0–16) sec; Brunner-Munzel test, T_39.6_ = 1.02, P > 0.05, effect size p̂ = 0.59]. Aged rats displayed less locomotor activity in the arena than young rats [total distance, mean (± standard deviation, SD): young, 14 (± 5.5) m; aged, 9.9 (± 3.2) m; Welch’s *t*-test, t_25.9_ = 3.04, P < 0.05, Hedge’s g = 0.81]. Young and aged rats were left untreated (Y-CON and A-CON groups) or challenged by two i.p. 1 mg/kg LPS injections given 24 and 48 h after the OFT (Y-LPS and A-LPS groups). In young rat groups, no significant difference was seen in the survival rate followed by the LPS challenge (Fig. 1b). There were significantly fewer survivors in the A-LPS group than the A-CON group [hazard ratio (HR) 0.13, 95% CI 0.03–0.46; log-rank test, z = 2.80, P < 0.05]. In the NORT test, the discrimination index of the A-LPS group was significantly decreased compared with those of the A-CON and Y-LPS groups (Fig. 1c). LPS significantly reduced locomotor activity in the A-LPS group (Fig. 1d). There were no significant differences between Y-CON and Y-LPS groups in the NORT.

### Aged sepsis-survivor rats showed NGAL elevations and iron dyshomeostasis in the hippocampus and cortex

**Figure 2:**
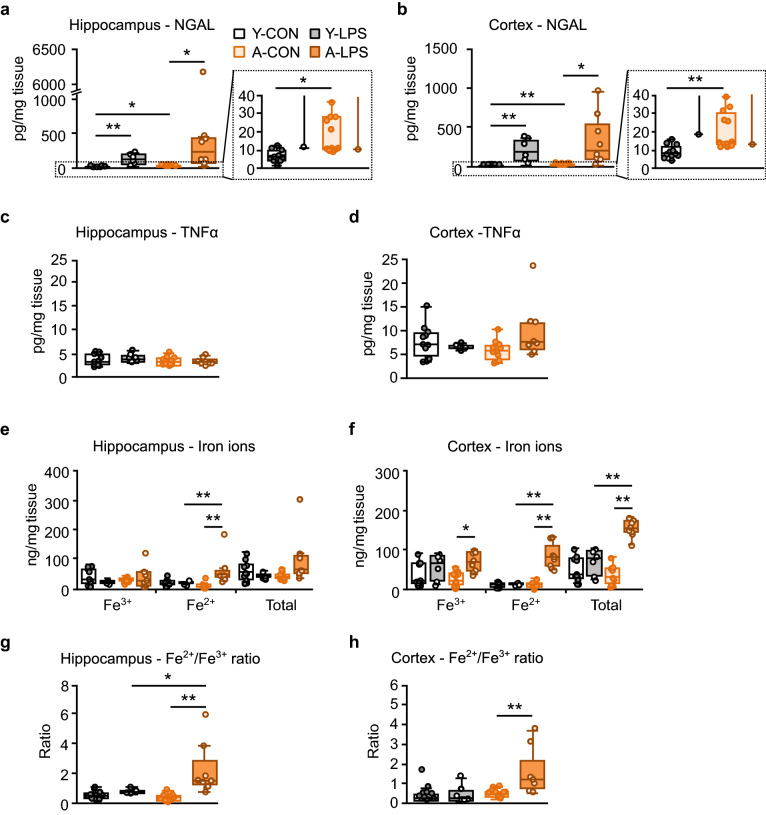
Aged sepsis-survivor rats showed neutrophil gelatinase-associated lipocalin (NGAL) elevations and dysregulation of iron homeostasis in the hippocampus and cortex. (**a**,**b**) The Y-LPS and A-LPS groups showed significant elevations of NGAL levels in the hippocampus and cortex. The hippocampal and cortical NGAL concentrations in the A-CON group were higher than those in the Y-CON group. (**c**,**d**) There were no significant changes in the hippocampal and cortical tumor necrosis factor α (TNFα) levels. (**e**) The A-LPS group displayed increases in the hippocampal Fe^2+^ levels compared with the A-CON group. (**f**) The concentration of Fe^3+^, Fe^2+^, and total iron ion in the cortex in the A-LPS group was higher than those in the A-CON group. (**g**,**h**) The hippocampal and cortical Fe^2+^/Fe^3+^ ratio of the A-LPS group was increased compared with those of the A-CON group. The data are presented as the median ± IQR. Y-CON, n = 11; Y-LPS, n = 6; A-CON, n = 11; A-LPS, n = 8. Permuted Brunner-Munzel test with Bonferroni correction, *P < 0.05, effect size p̂ ≥ 0.87, **P < 0.01, p̂ ≥ 0.90.

LPS significantly increased NGAL levels in the hippocampus and cortex in the Y-LPS and A-LPS groups (Fig. 2a,b). The A-CON group displayed higher hippocampal and cortical NGAL levels than the Y-CON group [hippocampus, median (IQR): Y-CON, 6.2 (4.2–9.6) pg/mg tissue; A-CON, 11.0 (10.0–28.0) pg/mg tissue; permutated Brunner-Munzel test, T = 4.60, P < 0.05, effect size p̂ = 0.87; cortex, median (IQR): Y-CON, 7.8 (6.5–12.1) pg/mg tissue; A-CON, 14.0 (12.5–30.3) pg/mg tissue; T = 6.08, P < 0.01, p̂ = 0.90]. No significant differences were observed in TNFα levels in the hippocampus or cortex (Fig. 2c,d). In the hippocampus (Fig. 2e), the concentration of Fe^2+^ in the A-LPS group was significantly higher than those of the A-CON and Y-LPS groups. The cortical Fe^3+^, Fe^2+^, and total iron concentration in the A-LPS group were significantly increased compared with those in the A-CON group (Fig. 2f). The ratio of hippocampal Fe^2+^ to Fe^3+^ in A-LPS group rats was higher than those in A-CON and Y-LPS group rats (Fig. 2g). The A-LPS group showed a higher cortical Fe^2+^/Fe^3+^ ratio than the A-CON groups (Fig. 2h).

### DFO pretreatment prevented LPS-caused object recognition impairment in aged rats

**Figure 3:**
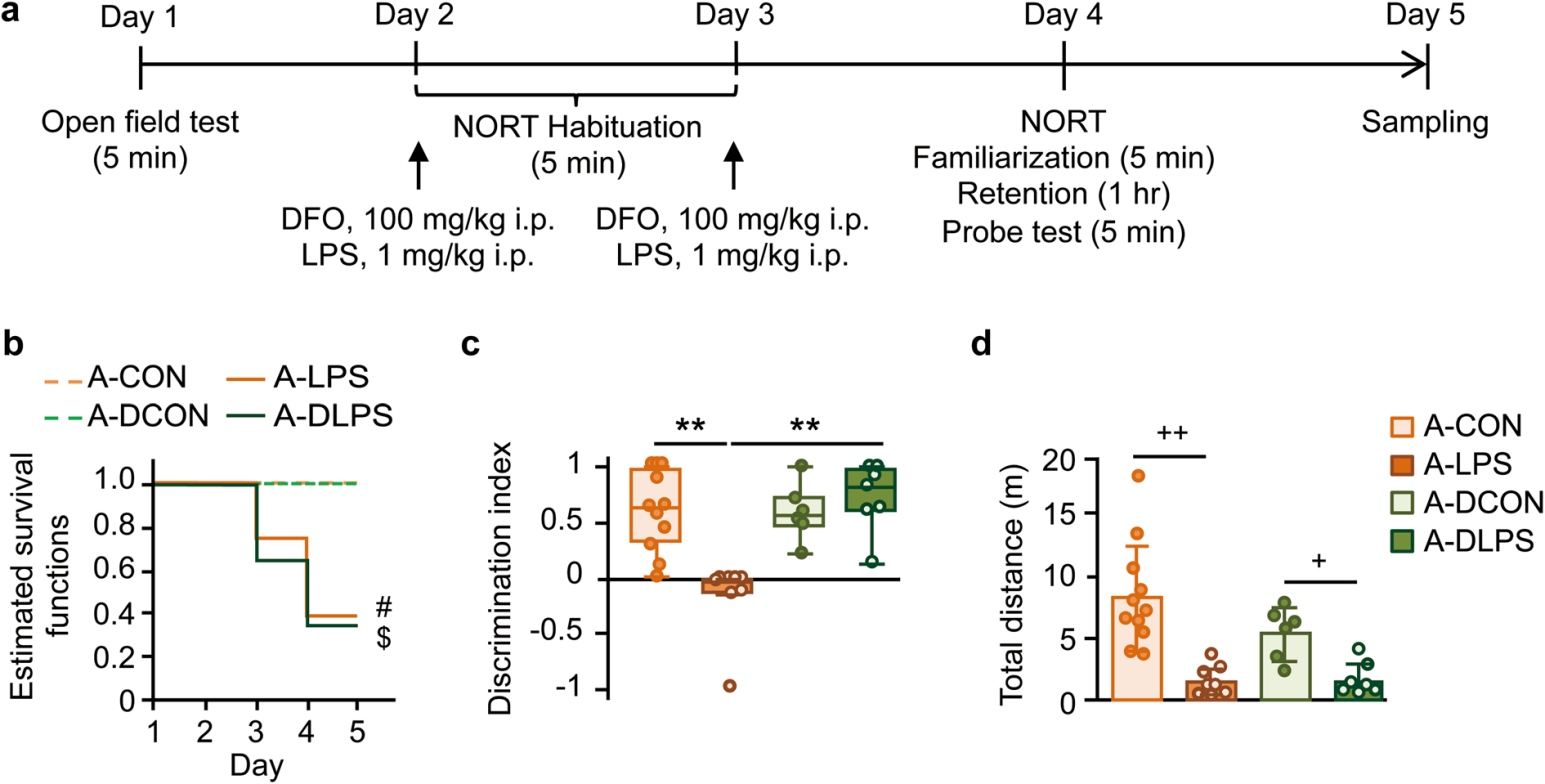
Deferoxamine (DFO) pretreatment blocked LPS-induced impairment of object discrimination in aged rats. (**a**) Aged rats were assigned to the 100 mg/kg DFO intraperitoneal treatment or 1 mg/kg LPS injection with DFO pretreatment group (A-DCON, n = 6, or A-DLPS, n = 20). DFO was administered 1 h before LPS injections. Details of the A-CON and A-LPS groups were as in Fig. 1. (**b**) The survival rate of the A-DLPS group was significantly lower than that of the A-DCON group. There was no significant difference between the survival rates of the A-LPS and A-DLPS groups. (**c**) In the NORT, A-DLPS group rats showed a higher discrimination index than A-LPS group rats. (**d**) DFO pretreatment did not attenuate the LPS-induced reduction of locomotion in the NORT. The behavioral data are presented as the median ± IQR or mean ± SD. A-CON, n = 11; A-LPS, n = 8; A-DCON, n = 6; A-DLPS, n = 7. Log-rank test with Bonferroni correction: ^#^P < 0.05, A-CON vs. A-LPS; ^$^P < 0.05, A-DCON vs. A-DLPS. Permuted Brunner-Munzel test with Bonferroni correction: **P < 0.01, effect size p̂ = 0.96. Welch’s ANOVA [F_3,14.1_ = 12.8, P < 0.001, ω^2^ = 0.66] and post-hoc Bonferroni corrected Welch’s *t*-test, ^+^P < 0.05, Hedge’s g = 1.97, ^++^P < 0.01, g = 2.08.

We next investigated whether DFO pretreatment attenuated LPS-induced lethal sepsis and object recognition deficit of aged rats (Fig. 3a). Another 26 rats were randomly assigned to the 100 mg/kg DFO injection (A-DCON, n = 6) or 1 mg/kg LPS with 100 mg/kg DFO preinjection (A-DLPS, n = 20) groups. There were no significant differences in baseline activity in the OFT between groups [time spent in the center area, median (IQR): aged-group, 4.0 (2.4–8.1) s; DFO-treated aged-group, 4.2 (2.1–7.7) s; Brunner-Munzel tests, T_53.3_ = 0.07, P > 0.05, effect size p̂ = 0.49; distance traveled, mean (± SD): aged-group, 9.9 (± 3.2) m; DFO-treated aged-group, 11 (± 2.9) m; Welch’s t-test, t_54.7_ = 1.8, P > 0.05, Hedge’s g = 0.48]. Pretreatment with DFO did not influence the survival rate after LPS challenge (A-DLPS vs. A-LPS, HR 0.78, 95% CI 0.29–2.10; log-rank test, z = 0.23, P > 0.05; Fig. 3b). DFO pretreatment blocked LPS-induced object discrimination impairment of the A-DLPS group in the NORT (Fig. 3c). Pretreatment with DFO did not influence LPS-induced decreases in locomotor activity (Fig. 3d).

### Pretreatment with DFO blocked iron ion accumulation and imbalance in the hippocampus and cortex in aged sepsis-survivor rats

**Figure 4:**
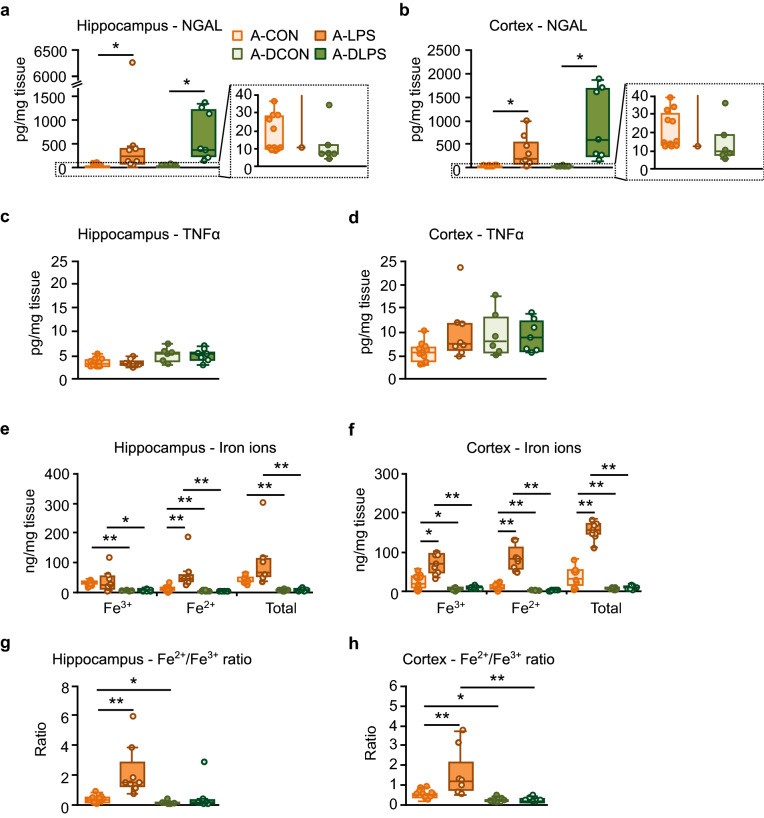
LPS-induced iron ion elevation and imbalance were blocked by pretreatment with DFO in aged sepsis-survivor rats. (**a**,**b**) A-DLPS rats showed NGAL level elevations in the hippocampus and cortex. (**c**,**d**) There were no significant differences in the hippocampal and cortical TNFα concentrations among groups. (**e–f**) The hippocampal and cortical iron ion levels in the A-DLPS group were significantly lower than those in the A-LPS group. (**g**,**h**) The A-DLPS group showed a lower Fe^2+^/Fe^3+^ ratio in the cortex but not in the hippocampus than the A-LPS group. DFO treatment significantly reduced each iron ion level and Fe^2+^/Fe^3+^ ratio in the hippocampus and cortex. The data are presented as the median ± IQR. A-CON, n = 11; A-LPS, n = 8; A-DCON, n = 6; A-DLPS, n = 7. Permuted Brunner-Munzel test with Bonferroni correction: *P < 0.05, effect size p̂ ≥ 0.89, **P < 0.01, p̂ ≥ 0.92.

DFO pretreatment did not suppress LPS-induced elevation of NGAL levels in the hippocampus and cortex (Fig. 4a,b). TNFα levels in the hippocampus and cortex were comparable among the groups (Fig. 4c,d). DFO injection reduced the levels of iron ions in the hippocampus and cortex (Fig. 4e,f). The hippocampal and cortical iron ion levels in the A-DLPS group were significantly lower than those in the A-LPS group. In the hippocampus, there was no significant difference between A-LPS and A-DLPS groups in the ratio of Fe^2+^ to Fe^3+^ (Fig. 4g). The cortical Fe^2+^/Fe^3+^ ratio in the A-DLPS group was significantly lower than that in the A-LPS group (Fig. 4h). There were no significant differences in each iron ion level and the Fe^2+^/Fe^3+^ ratio in the hippocampus or cortex between the A-DCON and A-DLPS groups.

### Hippocampal and cortical iron levels were negatively correlated with object recognition ability

**Figure 5:**
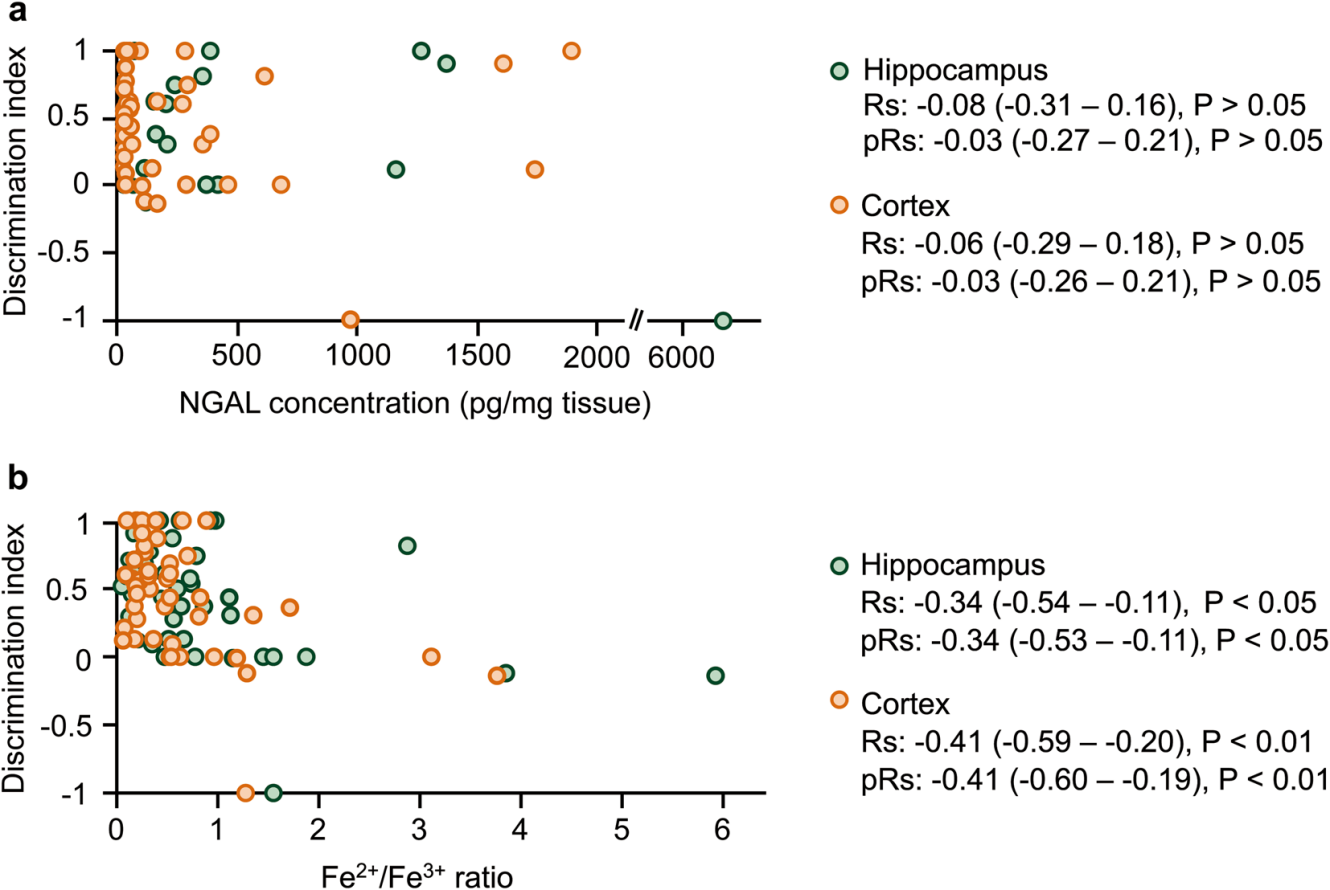
Object discrimination ability was negatively correlated with the Fe^2+^/Fe^3+^ ratio in the hippocampus and cortex. (**a**) Spearman correlation (Rs) and partial correlation (pRs) analysis revealed no significant relationship between the discrimination index and the hippocampal and cortical NGAL levels. (**b**) The Fe^2+^/Fe^3+^ ratio in the hippocampus and cortex exhibited a significant negative correlation with the discrimination index. The partial correlation analysis was controlled for Fe^2+^/Fe^3+^ ratio (**a**) or NGAL level (**b**). The coefficients are presented with (95% CI). n = 49.

The Spearman correlation and partial correlation analysis controlling for NGAL or Fe^2+^/Fe^3+^ ratio were performed to estimate the relationship between object recognition ability and brain NGAL or iron levels. There were no significant correlations between the discrimination index and NGAL level in the hippocampus and cortex (Fig. 5a). The Spearman correlation and partial correlation analysis controlling for NGAL revealed that the Fe^2+^/Fe^3+^ ratio in the hippocampus and cortex had a significant negative correlation with the discrimination index (Fig. 5b).

## Discussion

In this study, we investigated the effects of LPS-induced sepsis on object recognition and brain NGAL, TNFα, and iron ion levels in aged and young survivor rats. Young sepsis-survivor rats showed elevation of NGAL levels in the hippocampus and cortex. Aged sepsis-survivor rats displayed impairment of object recognition ability, elevations in the hippocampal and cortical NGAL levels, and dysregulation of iron ion levels. Pretreatment with the iron chelator DFO inhibited LPS-induced object recognition impairment and iron ion elevations. In addition, the Fe^2+^/Fe^3+^ ratio in the hippocampus and cortex was negatively correlated with object recognition ability. These results suggest that LPS induces cognitive dysfunction associated with dysregulation of iron metabolism in aged sepsis-survivor rats. Furthermore, DFO may prevent these LPS-induced deleterious effects.

In our study, dual LPS injection caused lethal sepsis but not induced hippocampal or cortical TNFα responses 48 h after the last injection in aged survivor rats. Preconditioning of sublethal-dose endotoxin exposure induces tolerance to subsequent endotoxin exposure, reducing TNFα response and mortality^21^. This discrepancy might be because of differences in the experimental procedures (doses and timing of LPS injections). Although we could not further investigate endotoxin tolerance in this study, we found that dual LPS challenge induced severe sepsis, NGAL elevation, and cognitive impairment in aged rats. Recently, increasing evidence that NGAL is involved in both neuroinflammation and neuroprotection has been obtained^22^. Upon exposure to inflammatory conditions, NGAL is predominantly synthesized and secreted by astrocytes in the brain^14^. Secreted NGAL stimulates surrounding astrocytes and microglia to become reactive, resulting in an amplified inflammatory response^12,13^. On the other hand, NGAL absence exacerbates proinflammatory cytokine and chemokine responses in the mouse CNS after systemic LPS challenge or experimental autoimmune encephalomyelitis^15,16^. These studies indicate that NGAL might serve as a neuroprotective factor in response to neuroinflammation. However, this study provided no direct evidence that NGAL exerts inflammatory or protective effects or both.

In pathological conditions, iron accumulation and ferric/ferrous ion imbalance produce reactive oxygen species like highly reactive hydroxyl radicals by Fenton reaction, resulting in apoptosis, necrosis, autophagy, and ferroptosis^20,23^. Indeed, patients with neurodegenerative diseases, including Alzheimer’s disease and Parkinson’s disease, show iron accumulation in the cortex and hippocampus^24,25^. A recent study revealed that LPS induces cognitive deficits and iron accumulation in the hippocampi and cortices of aged mice by upregulating microglial heme oxygenase-1 (HO-1) expression^26^. Overexpression of HO-1 in older adults causes cognitive decline and aggregation of Aβ and Tau along with iron accumulation^27,28^. LPS increases iron accumulation with upregulation of divalent metal transporter 1 (DMT1) expression in neurons and microglia^29^. These reports support our present findings that increases in Fe^2+^ levels and Fe^2+^/Fe^3+^ imbalances in the hippocampus and cortex might contribute to cognitive disturbance in LPS-injected aged rats.

In the present study, pretreatment with DFO blocked LPS-induced increases in Fe^2+^ levels, iron dysmetabolism, and cognitive deficits in aged rats. Partly consistent with our findings, DFO treatment for six consecutive days can prevent neuroinflammation, iron overload, and sickness behaviors^26^. DFO has a high affinity for Fe^3+^ and might chelate them in the brain following disruption of the BBB by LPS^30,31^. DFO-induced Fe^3+^ chelation reduces the cytosolic labile iron pool (LIP), attenuates the expression of both DMT1 and the iron storage ferritin, and promotes the expression of the iron exporter ferroportin 1 after LPS treatment^26,32^. In addition, the reversible Fe^3+^ to Fe^2+^ transition might compensate for decreased Fe^3+^ levels through the cytosolic LIP^33^. Indeed, compared with aged survivors, DFO-pretreated aged rats showed reductions in total iron levels and the Fe^2+^/Fe^3+^ ratio in the hippocampus and cortex. Therefore, iron chelation might contribute to iron homeostasis in the hippocampus and cortex after endotoxin challenge to maintain cognitive function.

There were several limitations of this study. First, we found that aging increased approximately twofold NGAL levels in the cortex and hippocampus. A recent human and mouse plasma proteome study reveals that plasma NGAL levels show an aging-related exponential increase^34^, suggesting that NGAL might serve as an aging predictor of the CNS; however, the mechanisms remain unclear. Second, based on our previous study^18^, the present study assumed that DFO pretreatment would attenuate LPS-induced NGAL elevation and cognitive deficits. However, our present results have supported the finding that NGAL elevation in the CNS during LPS-based sepsis did not affect cognitive impairments^22^. Unexpectedly, LPS injections following DFO pretreatments caused a greater brain NGAL level elevation than LPS injections alone. Although iron-chelated DFO has anti-microbial potential, some gram-negative and -positive bacteria can use DFO for efficient iron uptake and growth^35,36^. These findings suggest that DFO pretreatment had promoted pathogenic bacterial growth in aged rat body cavity even if under a specific pathogen-free environment, probably leading to enhance immune response and sepsis. Thus, DFO-induced iron depletion might prime NGAL responsiveness as an anti-bacterial effector or iron donor; however, whether LPS-induced NGAL elevation exerts pathogenic, protective, or no roles in the CNS via iron-endocytosis or -exocytosis remains unknown. Furthermore, the dose of DFO 32 mg/kg/day, equivalent to rat dose 200 mg/kg^37^, improved outcomes of patients with intracerebral hemorrhage at day-180^38^. In the aged rat model of intracerebral hemorrhage, DFO 100 mg/kg (human-equivalent dose, 16 mg/kg/day^37^), the same amount used in this study, improved neurological and functional recovery^39^. These clinical trials and experimental studies indicate that DFO might have protective effects on cognitive function; however, additional studies are needed to validate the safety and effectiveness of DFO to sepsis-associated cognitive dysfunction. Finally, we could not investigate the effects of DFO on brain HO-1 expression and other cognitive-behavioral tests such as the object location test, contextual fear conditioning, and various operant tasks in this study. Further behavioral and biochemical studies are required to provide a comprehensive mechanism of sepsis-induced cognitive dysfunction, including NGAL, HO-1, and other iron regulator proteins.

In conclusion, we have demonstrated that iron homeostasis in the cortex and hippocampus contributes to maintenance of object recognition after LPS-induced sepsis in aged rats. Although further study is required to improve the survival rate, our study provides new insights into the potential of pharmacological iron chelators as therapeutic targets for sepsis-associated cognitive deficits, including SAE.

## Methods

### Animals

Male Sprague–Dawley rats aged 1.5–3 months (n = 19, 180–440 g) and 12–18 months (n = 57, 580–900 g) were used for behavioral and biochemical analysis. The animals were group-housed at 24 ± 2 °C under a 12-h light–dark cycle (lights on at 8:00 a.m.), and food and water were available ad libitum. All experiments were carried out in accordance with the ARRIVE Guideline and the guidelines for animal research issued by the Hirosaki University Graduate School of Medicine. This study was approved by the Animal Research Committee of Hirosaki University (approval number M19019). All experimental analyses were performed by the observers blinded and automatic analysis software. All efforts were made to minimize the number of animals used and their suffering.

## Drugs

Rats in the LPS groups were intraperitoneally (i.p.) injected with 1 mg/kg of LPS (*E. coli* O111:B4; Wako Pure Chemical Industries, Osaka, Japan) after two consecutive daily habituation sessions for the novel object recognition test. Another groups of aged rats were i.p. treated with 100 mg/kg of DFO (Novartis Pharmaceutical Corporation, Tokyo, Japan) 1 h before LPS administration or treated with DFO^39^.

### Behavioral experiment protocol

An experimental protocol based on and modified from previous studies was used to investigate the effects of LPS on cognitive function^40,41^. Rats were randomly assigned to four groups: the aged control (A-CON); aged 1 mg/kg LPS injection (A-LPS); young control (Y-CON); and young 1 mg/kg LPS (Y-LPS) groups (Fig. 1a). Other aged rats were randomly assigned to the 100 mg/kg DFO injection (A-DCON) or 1 mg/kg LPS with 100 mg/kg DFO preinjection (A-DLPS) groups (Fig. 3a).

### Open field test (OFT)

We performed the OFT to confirm the effects of age on locomotor activity and anxiety. The behavior of the rats in an OFT arena (60 × 60 × 60 cm, 50 lx) was recorded for 5 min using the CaptureStar video tracking system (CleverSys, Reston, VA, USA). Horizontal locomotor activity [total distance traveled (m)] and the time spent in the center area (s) were automatically analyzed with TopScan behavioral analysis software (CleverSys).

### Novel object recognition test (NORT)

Rats were habituated to a NORT arena (60 × 60 × 60 cm, 50 lx) not containing objects for 5 min for two consecutive days. Twenty-four hours after the last habituation session, the animals were subjected to the NORT. Each rat was introduced to the test arena, which contained two reference objects, and allowed to explore for 5 min freely (familiarization session). After a 1-h retention interval, the animals were returned to the same test arena containing a familiar object and novel one and allowed to explore for 5 min (probe test session). The objects were colored brick towers (5 × 5 × 10 cm), white Styrofoam balls (10 cm diameter), or grey steel boxes (3 × 8 × 8 cm). These objects were randomly used as familiar or novel without duplication. During each session, exploratory behavior was recorded with the CaptureStar system (CleverSys). The total distance traveled (m) and time spent exploring each object (s) in the probe test were automatically analyzed by TopScan software (CleverSys). The following formula was used to assess novel object recognition ability during the NORT probe test session: discrimination index = [novel object contact (s) − familiar object contact (s)]/total object contact time (s).

### Biochemical assay for NGAL and TNFα

Rats were sacrificed by rapid decapitation 24 h after the NORT, and the hippocampi and cortices were collected. Each brain sample was homogenized in ice-cold phosphate-buffered saline. The centrifuged supernatants were stored at − 20 or – 80 °C until further analysis. NGAL levels in the hippocampus and cortex were measured using an enzyme-linked immunosorbent assay kit (NGAL ELISA Kit, KIT046, BioPort Diagnostics, Hellerup, Denmark). The assay detection limit was 0.5 pg/ml, and the intra- and interassay coefficients of variance were 4% and 12%, respectively. The hippocampal and cortical TNFα levels were also determined by an ELISA kit (TNF-α ELISA kit CSB-E11987r, Cusabio Technology, Wuhan, China). The detection limit of the assay was 1.56 pg/ml, and the intra- and interassay coefficients of variance were < 8% and < 10%, respectively. Data were obtained using the Bio-Rad iMark™ Microplate Reader (Bio-Rad Laboratories, Hercules, CA, USA).

### Iron ion level assay

Total iron and Fe^2+^ levels in the hippocampus and cortex were measured using an iron assay kit (Iron Assay Kit ab83366, Abcam, Tokyo, Japan). According to the instructions, the brain samples were homogenized in assay buffer and centrifuged. For Fe^2+^ level assay, Fe^2+^ reacted with 3-(2-pyridyl)-5,6-bis(2-(5-furylsulfonic acid))-1,2,4-triazine (Ferene-S) was measured at 593 nm. Total iron ions were detected at 593 nm following the reduction of Fe^3+^ to Fe^2+^ using an iron reducer provided by the assay kit^42^. Colorimetric data were acquired using the microplate reader (Bio-Rad Laboratories). The assay detection limit was 8 μM. The Fe^3+^ level was calculated by subtracting the Fe^2+^ level from the total iron ion level.

### Statistical analysis

The sample size (estimated minimal N/group = 5) to achieve a power (1-β error probability) of 0.8, α error probability of 0.05, and effect size of 0.94 was determined by G*Power 3.1.9.7 (Faul F, University of Kiel, Kiel, Germany) based on the NORT study by Kawano et al.^40,43^. The Shapiro–Wilk test was used to estimate the normality of the data^44^. Based on the normality, data are presented as the mean (standard deviation, SD) or median (inter-quartile range, IQR) with individual data points^45^. The log-rank test for Kaplan–Meier analysis with Bonferroni correction was used to assess the survival data between groups^46^. The two-tailed Welch’s *t*-test, Welch’s analysis of variance (ANOVA) followed by the post hoc *t*-test, Brunner-Munzel test, or Brunner-Munzel permutation test was used to determine the significance of differences between groups^47^. P < 0.05 was considered significant. For multiple comparisons, each P-value was adjusted to < 0.05 by the Bonferroni correction. The effect size was estimated using Hedge’s g, ω^2^, or p̂^47,48^. The Spearman correlation analysis and partial correlation analysis controlling for NGAL level or Fe^2+^/Fe^3+^ ratio in each brain region were performed to evaluate the relationships between the discrimination index and NGAL level or Fe^2+^/Fe^3+^ ratio. Statistical analyses were performed with R version 4.0.5 (R Core Team, R Foundation for Statistical Computing, Vienna, Austria) and MATLAB 2020b (The MathWorks, Natick, MA, USA).

## Data Availability

The datasets used during the current study are available from the corresponding author on reasonable request.

## References

[1] Singer M (2016). The third international consensus definitions for sepsis and septic shock (sepsis-3). JAMA.

[2] Iwashyna TJ, Ely EW, Smith DM, Langa KM (2010). Long-term cognitive impairment and functional disability among survivors of severe sepsis. JAMA.

[3] Semmler A (2013). Persistent cognitive impairment, hippocampal atrophy and EEG changes in sepsis survivors. J. Neurol. Neurosurg. Psychiatry.

[4] Kuperberg SJ, Wadgaonkar R (2017). Sepsis-associated encephalopathy: The blood–brain barrier and the sphingolipid rheostat. Front. Immunol..

[5] Godbout JP (2005). Exaggerated neuroinflammation and sickness behavior in aged mice after activation of the peripheral innate immune system. FASEB J..

[6] Henry CJ, Huang Y, Wynne AM, Godbout JP (2009). Peripheral lipopolysaccharide (LPS) challenge promotes microglial hyperactivity in aged mice that is associated with exaggerated induction of both pro-inflammatory IL-1β and anti-inflammatory IL-10 cytokines. Brain Behav. Immun..

[7] Hou X (2020). The value of neutrophil gelatinase-associated lipocalin and citrullinated alpha enolase peptide-1 antibody in diagnosis, classification, and prognosis for patients with sepsis. Medicine.

[8] Ferreira AC (2015). From the periphery to the brain: Lipocalin-2, a friend or foe?. Prog. Neurobiol..

[9] Flo TH (2004). Lipocalin 2 mediates an innate immune response to bacterial infection by sequestrating iron. Nature.

[10] Ip JPK, Noçon AL, Hofer MJ, Lim SL, Müller M, Campbell IL (2011). Lipocalin 2 in the central nervous system host response to systemic lipopolysaccharide administration. J. Neuroinflamm..

[11] Dekens DW (2018). Lipocalin 2 contributes to brain iron dysregulation but does not affect cognition, plaque load, and glial activation in the J20 Alzheimer mouse model. J. Neuroinflamm..

[12] Lee S (2007). A dual role of lipocalin 2 in the apoptosis and deramification of activated microglia. J. Immunol..

[13] Lee S (2009). Lipocalin-2 is an autocrine mediator of reactive astrocytosis. J. Neurosci..

[14] Mesquita SD (2014). Lipocalin 2 modulates the cellular response to amyloid beta. Cell Death Differ..

[15] Berard JL (2012). Lipocalin 2 is a novel immune mediator of experimental autoimmune encephalomyelitis pathogenesis and is modulated in multiple sclerosis. Glia.

[16] Kang SS (2018). Lipocalin-2 protects the brain during inflammatory conditions. Mol. Psychiatry.

[17] Xing C (2014). Neuronal production of lipocalin-2 as a help-me signal for glial activation. Stroke.

[18] Furukawa T (2017). Chronic diazepam administration increases the expression of Lcn2 in the CNS. Pharmacol. Res. Perspect..

[19] Brandtner A (2020). Linkage of alterations in systemic iron homeostasis to patients’ outcome in sepsis: A prospective study. J. Intensive Care.

[20] Ward RJ, Zucca FA, Duyn JH, Crichton RR, Zecca L (2014). The role of iron in brain ageing and neurodegenerative disorders. Lancet Neurol..

[21] López-Collazo E, del Fresno C (2013). Pathophysiology of endotoxin tolerance: Mechanisms and clinical consequences. Crit. Care..

[22] Olson B (2021). Chronic cerebral lipocalin 2 exposure elicits hippocampal neuronal dysfunction and cognitive impairment. Brain Behav. Immune..

[23] Hota KB, Hota SK, Srivastava RB, Singh SB (2012). Neuroglobin regulates hypoxic response of neuronal cells through Hif-1α- and Nrf2-mediated mechanism. J. Cereb. Blood Flow Metab..

[24] Ayton S (2017). Cerebral quantitative susceptibility mapping predicts amyloid-β-related cognitive decline. Brain.

[25] Thomas GEC (2020). Brain iron deposition is linked with cognitive severity in Parkinson’s disease. J. Neurol. Neurosurg. Psychiatry..

[26] Fernández-Mendívil C (2021). Protective role of microglial HO-1 blockade in aging: Implication of iron metabolism. Redox Biol..

[27] Fernández-Mendívil C, Arreola MA, Hohsfield LA, Green KN, López MG (2020). Aging and progression of beta-amyloid pathology in alzheimer’s disease correlates with microglial heme-oxygenase-1 overexpression. Antioxidants.

[28] Hui Y (2011). Long-term overexpression of heme oxygenase 1 promotes tau aggregation in mouse brain by inducing tau phosphorylation. J. Alzheimers Dis..

[29] Urrutia P (2013). Inflammation alters the expression of DMT1, FPN1 and hepcidin, and it causes iron accumulation in central nervous system cells. J. Neurochem..

[30] Banks WA (2015). Lipopolysaccharide-induced blood-brain barrier disruption: Roles of cyclooxygenase, oxidative stress, neuroinflammation, and elements of the neurovascular unit. J. Neuroinflamm..

[31] Eybl V, Kotyzová D, Kolek M, Koutenský J, Nielsen P (2002). The influence of deferiprone (L1) and deferoxamine on iron and essential element tissue level and parameters of oxidative status in dietary iron-loaded mice. Toxicol. Lett..

[32] Zhang XY, Cao JB, Zhang LM, Li YF, Mi WD (2015). Deferoxamine attenuates lipopolysaccharide-induced neuroinflammation and memory impairment in mice. J. Neuroinflamm..

[33] Belaidi AA, Bush AI (2016). Iron neurochemistry in Alzheimer’s disease and Parkinson’s disease: Targets for therapeutics. J. Neurochem..

[34] Lehallier B (2019). Undulating changes in human plasma proteome profiles across the lifespan. Nat. Med..

[35] Kim CM, Park YJ, Shin SH (2007). A widespread deferoxamine-mediated iron-uptake system in *Vibrio vulnificus*. J. Infect. Dis..

[36] Arifin AJ, Hannauer M, Welch I, Heinrichs DE (2014). Deferoxamine mesylate enhances virulence of community-associated methicillin resistant *Staphylococcus aureus*. Microbes Infect..

[37] Selim M (2011). Safety and tolerability of deferoxamine mesylate in patients with acute intracerebral hemorrhage. Stroke.

[38] Selim M (2019). Deferoxamine mesylate in patients with intracerebral haemorrhage (i-DEF): A multicentre, randomised, placebo-controlled, double-blind phase 2 trial. Lancet Neurol..

[39] Okauchi M, Hua Y, Keep RF, Morgenstern LB, Xi G (2009). Effects of deferoxamine on intracerebral hemorrhage-induced brain injury in aged rats. Stroke.

[40] Kawano T (2015). Impact of preoperative environmental enrichment on prevention of development of cognitive impairment following abdominal surgery in a rat model. Anesthesiology.

[41] Nikaido Y (2016). Cis-3-Hexenol and trans-2-hexenal mixture prevents development of PTSD-like phenotype in rats. Behav. Brain Res..

[42] Leal SM (2013). Targeting iron acquisition blocks infection with the fungal pathogens *Aspergillus fumigatus* and *Fusarium oxysporum*. PLOS Pathog..

[43] Faul F, Erdfelder E, Lang AG, Buchner A (2007). G*Power 3: A flexible statistical power analysis program for the social, behavioral, and biomedical sciences. Behav. Res. Methods.

[44] Ö̈ner M, Kocakoç İD (2017). JMASM 49: A compilation of some popular goodness of fit tests for normal distribution: Their algorithms and MATLAB codes (MATLAB). J. Mod. Appl. Stat. Methods..

[45] Borau, C. BoxPlotPro. MATLAB Central File Exchange. Retrieved May 14, 2021 from https://www.mathworks.com/matlabcentral/fileexchange/88733-boxplotpro (2021).

[46] Cardillo, G. LogRank: Comparing survival curves of two groups using the log rank test. MATLAB Central File Exchange. Retrieved May 14, 2021 from http://www.mathworks.com/matlabcentral/fileexchange/22317 (2021).

[47] Konietschke F, Placzek M, Schaarschmidt F, Hothorn L (2015). nparcomp: An R software package for nonparametric multiple comparisons and simultaneous confidence intervals. J. Stat. Softw..

[48] Ben-Shachar M, Lüdecke D, Makowski D (2020). effectsize: Estimation of effect size indices and standardized parameters. J. Open Source Softw..

